# Formulation of Propolis Phenolic Acids Containing Microemulsions and Their Biopharmaceutical Characterization

**DOI:** 10.1155/2016/8175265

**Published:** 2016-11-03

**Authors:** Modestas Žilius, Kristina Ramanauskienė, Vaida Juškaitė, Vitalis Briedis

**Affiliations:** Department of Clinical Pharmacy, Lithuanian University of Health Sciences, Sukilėlių Street 13, LT-50161 Kaunas, Lithuania

## Abstract

Microemulsions (MEs) were formulated using PEG-8 caprylic/capric glycerides and ethanolic propolis extracts. Characterization of MEs was performed by determining mean droplet size, polydispersity index, stability under varying external factors, and formulation effect on delivery of phenolic compounds into the skin* ex vivo*. Essential oils were included into the formulations of MEs and their influence on physical characteristics of the nanostructured systems as well as penetration into epidermis and dermis were evaluated. The droplet size, their distribution, and stability of the formulated MEs were not affected. Presence of essential oils in the formulation increased penetration of phenolic compounds in general, but only the amount of ferulic acid increased significantly. Mean droplet size increased with increase of oily phase amount, suggesting that phenolic compounds and components of essential oils were not modifying the formation of the interphase film composition and/or structure. Phenolic compounds were predominantly located in the lipid phase of the MEs thus minimizing their availability at the surface of the skin.

## 1. Introduction

Increasing understanding of risks affecting human skin and threats of resultant pathology is supporting search for new approaches and formulating innovative products to protect the outmost cells of human organism. External stress factors and ultraviolet radiation in particular can cause damage of the exposed skin cells resulting in oxidative stress, premature aging, and skin cancers [1]. The association between skin ageing and increased oxidative stress is clarified by the fact that skin-ageing changes could cause elevated levels of oxidative stress. It should be emphasized that increased oxidative stress may cause skin ageing, and skin ageing and elevated oxidative stress levels may both indicate existence of some underlying general cause [2]. Topically applied sunscreen products can protect the skin from the harmful effects of UV radiation. The possibility to incorporate naturally occurring substances in sunscreen formulations is becoming an actual topic as those secondary metabolites typically demonstrate antioxidant and UV-absorbing ability. It is determined that phenolic acids, nonflavonoids, and flavonoids can act as UV blockers reducing inflammation, oxidative stress, and DNA damage [3].

Scientific evaluation of propolis and its products demonstrates antioxidant, antibacterial, antiviral, antifungal, anti-inflammatory, antitumoral, and wound healing properties of propolis; it stimulates tissue regeneration, wound healing, suppresses pain, and tumefaction [4–6]. Propolis is commonly used as a liquid extract in cosmetic and medicinal preparations because of its antiseptic, anti-inflammatory, and anesthetic properties. Complex varying qualitative and quantitative composition of propolis extracts is a recognized challenge in standardizing propolis products. Phenolic acids and their esters, flavonoids, aromatic alcohols and aldehydes, and terpenes are basic components of different origin propolis and their presence defines quality of propolis products as well as biological activity and possible application [7, 8]. Efficacy of topically applied products is resulting from ability of active components to diffuse into the skin from the applied carrier system. The intensity and the rate of skin penetration by propolis extract components depend on the physicochemical properties of individual compounds and on the structure and composition of the carrier system. The limited ability of propolis polyphenolic compounds (vanillic, caffeic, *p*-coumaric, ferulic acids, and vanillin) to penetrate into the skin has been determined; thus the application of could be considered as potential solution for achieving improved delivery of propolis antioxidant components into skin [9].

Cosmeceuticals are developed to enhance the health and beauty of skin and are classified as a category of products positioned between cosmetics and pharmaceuticals [10]. Protective cosmeceutical formulations should be both efficient and acceptable for consumer. The protective potential of biologically active components of propolis products could be enhanced by development of efficient delivery systems to transport required quantity of protecting compounds to the right site in the skin. Microemulsions are considered as promising carrier system for cosmetic active ingredients as they perform as efficient solubilizers for hydrophilic and lipophilic ingredients with high encapsulation capacity, improving product efficiency, stability, and appearance. They offer good cosmetic qualities and high hydration properties thus possibly enhancing skin penetration which may emphasize their importance in topical products [11]. Microemulsions are optically isotropic systems containing hydrophilic, lipophilic phases, and a mixture of surfactants. Thermodynamically stabile and transparent microemulsions are easily formulated systems with high diffusion and absorption rates. The low interfacial tensions of microemulsions provide excellent wetting properties ensuring good contact between formulations and the skin. The ingredients of microemulsions can effectively overcome the diffusion barrier and permeate the stratum corneum of the skin, offering efficient dermal and transdermal drug delivery [12]. The average droplet diameter in microemulsions could be in the range from 10 to 140 nm thus exhibiting properties of nanostructured systems. Appropriate selection of components for formulation of microemulsions can guarantee its biocompatibility, nontoxicity, and clinical acceptability.

The objective of this study was to formulate microemulsions containing propolis extracts and evaluate their physicochemical characteristics, stability, and ability to deliver propolis phenolic compounds into the skin* ex vivo*.

## 2. Materials and Methods

### 2.1. Chemicals and Reagents

Raw propolis was obtained from UAB Medicata Filia (Vilnius, Lithuania). Acetonitrile (Chromasolv) and acetic acid (glacial) were gradient grade for HPLC, ≥99.8% pure quality (Sigma-Aldrich Chemie GmbH, Steinheim, Germany). Ultrapure water was produced filtering purified water through the Millipore Simplicity HPLC grade water preparation cartridge (Bedford, USA). PEG-8 caprylic/capric glycerides (Labrasol) were purchased from Gattefosse (Saint-Priest, France); ethanol from AB “Vilniaus degtinė”, Vilnius, Lithuania; isopropyl myristate, synthesis grade from Scharlab SL, Barcelona, Spain; sodium chloride from Carl Roth GmbH, Karlsruhe, Germany; and sodium azide from POCh, Gliwice, Poland.

### 2.2. Ethanolic and Aqueous Propolis Extract

Phenolic compounds from raw propolis were extracted using 96% ethanol or purified water at a material-to-solvent ratio of 1 : 10 (w/v), stirring on a hotplate magnetic stirrer WiseStir MSH-20D (Wertheim, Germany) for 1 hour and at 70°C temperature (aqueous propolis extract). Produced ethanolic or aqueous propolis extract was filtered using Buchner vacuum filtration system.

### 2.3. Propolis Samples Analysis by High-Performance Liquid Chromatography

Phenolic acids (*p*-coumaric, ferulic, caffeic, and vanillic acids) and vanillin were quantified in propolis extract and samples using Agilent 1260 Infinity capillary LC (Agilent Technologies, Inc., Santa Clara, CA, USA) with Agilent diode array detector (DAD) and applying validated HPLC method: C18 column (150 × 0.5 mm, 5 *μ*m particle size); the linear elution gradient from 1 to 21% of solvent A (acetonitrile) in B (0.5% (v/v) acetic acid in ultrapure water) 25 min; the injection volume was 0.2 *μ*L, the flow rate was 20 *μ*L/min, and the column temperature was 25°C. The integration of phenolic compounds peaks was performed at 290 nm [9].

### 2.4. The Construction of Pseudoternary Phase Diagrams

**Figure 1:**
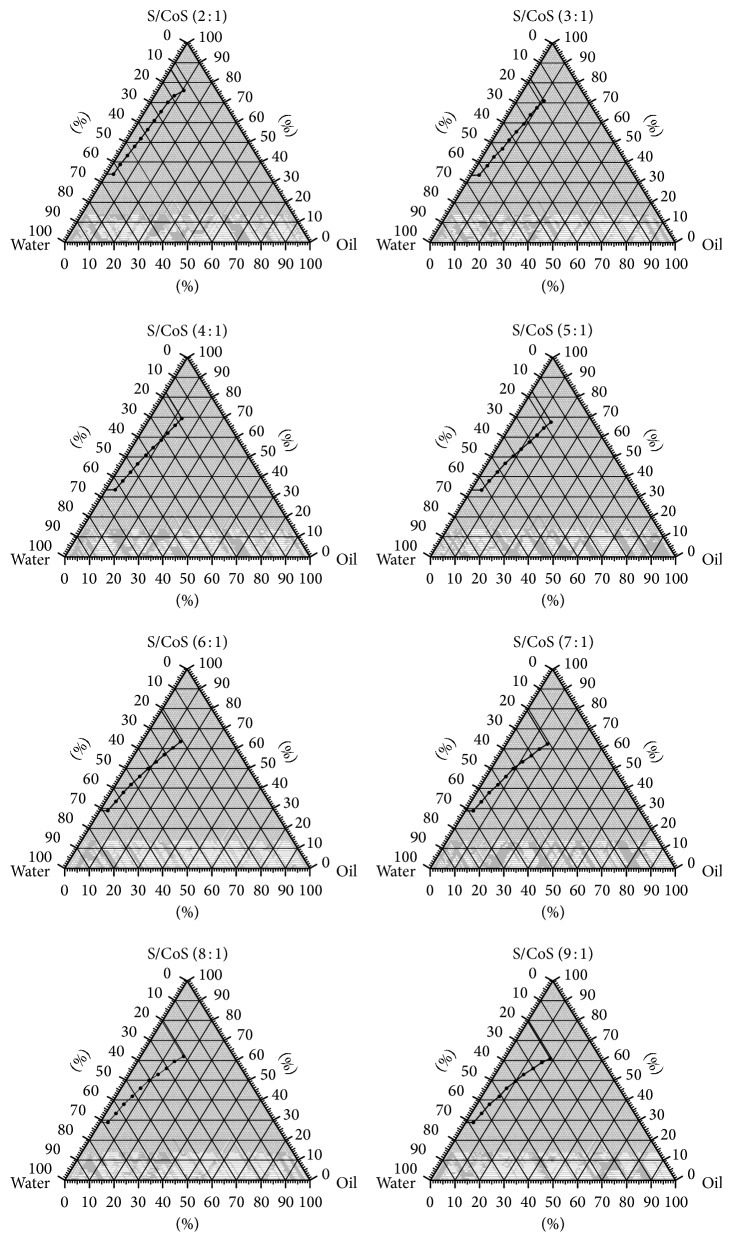
Pseudoternary phase diagrams of oil-in-water microemulsions.

Labrasol, 96% ethanol, isopropyl myristate, and purified water were selected as surfactant, cosurfactant, and oily and aqueous phase, respectively, for the construction of pseudoternary phase diagrams. The oil titration method was used for the production of the oil-in-water (o/w) microemulsions [13, 14]. The ratios of surfactant and cosurfactant in the microemulsions were 2 : 1, 3 : 1, 4 : 1, 5 : 1, 6 : 1, 7 : 1, 8 : 1, and 9 : 1. First, purified water (15–70%), surfactant, and cosurfactant (30–85% of mixture) were mixed stirring on a hotplate magnetic stirrer WiseStir MSH-20D at room temperature. Then oil was added by drops under stirring when the samples appeared as cloudy liquids (the limit of the o/w microemulsion). The regions of oil-in-water microemulsions were plotted at the pseudoternary phase diagrams (Figure 1).

### 2.5. Formulation of the Microemulsions Containing Ethanolic Propolis Extract

The same percentage composition of the o/w microemulsion was selected from the pseudoternary phase diagrams: 5% of oil phase, 25% of aqueous phase, and 70% of a mixture of surfactant and cosurfactant in different ratios (2 : 1, 3 : 1, 4 : 1, 5 : 1, 6 : 1, 7 : 1, 8 : 1, and 9 : 1). Purified water, labrasol (surfactant), ethanolic propolis extract (cosurfactant), and isopropyl myristate were mixed when microemulsions appeared as clear liquids (Table 1).

### 2.6. Thermodynamic Stability Studies of Propolis Microemulsions

Thermodynamic stability of o/w propolis microemulsions was evaluated performing centrifugation test and heating-cooling and freeze-thaw cycles test [15]. The microemulsions were centrifuged at 3500 rpm for 30 min. The microemulsions were stored at 4°C, 20°C, 32°C, and 45°C temperature for not less than 48 hours during heating-cooling cycle and at −21°C, 4°C, and 25°C temperature for not less than 48 hours during freeze-thaw cycle.

### 2.7. Physical Characterization of O/W Microemulsions

The droplet size, standard deviation, and polydispersity index (PDI) of o/w microemulsions were measured using Zetasizer Nano ZS particle size analyzer (Malvern, UK) [16]. The microemulsions pH was determined using pH-meter 766 (Knick, Germany); conductivity was determined using conductivity meter (Cond 3110 SET 1, Germany); viscosity was determined using Vibro-Viscometer SV-10 (I&D Company, Limited, Japan).

### 2.8. *Ex Vivo* Skin Penetration Study

Caucasian women (age range of 25–40 years) abdominal skin was obtained from Department of Plastic and Reconstructive Surgery, Hospital of Lithuanian, University of Health Sciences, after cosmetic surgery. Kaunas Region Bioethical Committee has approved the use of human skin for transdermal penetration studies.* Ex vivo *skin penetration studies (*n* = 6) were performed using Bronaugh type flow-through diffusion cells with full-thickness human skin. The efficient diffusion area in the cells was 0.64 cm^2^. The diffusion cells were placed on the metallic heating block maintaining 37°C temperature by a Grant TC120 thermostated circulating water bath (Grant Instruments Ltd., Cambridge, Great Britain). The acceptor medium (0.9% NaCl solution with 0.005% NaN3) was circulated underneath the skin samples maintaining 0.6 mL/min of circulation rate by Masterflex L/S peristaltic pump with multichannel pump head (Cole-Parmer Instrument Co., IL, USA). The infinite dose of the o/w propolis microemulsion was applied on the outer human skin side surface, and the diffusion cells were covered with aluminum foil. After 24 hours, microemulsions were removed from the human skin surface. The skin samples (0.64 cm^2^) were trimmed off removing the outer residuals. Epidermis was separated from dermis applying dry heat separation method [9, 17] and they were separately extracted with a mixture of methanol and deionized water (1 : 1) under sonication.

### 2.9. Statistical Analysis

Statistical analysis of experimental data was performed using SPSS software (version 19.0). Mann-Whitney *U* test and one-way ANOVA (Tukey's honestly significant difference criterion) were used for data analysis. Correlation analysis was performed applying Spearman's rank coefficient.

## 3. Results and Discussion

The concentrations of phenolic acids and vanillin were determined in the ethanolic and aqueous propolis extract (Table 2). The total concentration of phenolic acids and vanillin (2472.7 ± 24.6 *µ*g/mL) in ethanolic propolis extract was up to 7.5-fold higher comparing with that in aqueous propolis extract (330.5 ± 8.9 *µ*g/mL). Ethanolic propolis extract was used as an active component and cosurfactant for the formulation of microemulsions (MEs).

As presented in the constructed pseudoternary phase diagrams (Figure 1), the composition of MEs containing 5% of oily phase, 25% of aqueous phase, and 70% of a mixture of surfactant and cosurfactant in ratios 2 : 1, 3 : 1, 4 : 1, 5 : 1, 6 : 1, 7 : 1, 8 : 1, and 9 : 1 resulted in stable MEs and was selected for further development. Ethanol was used as cosurfactant in formulating o/w MEs during initial testing and obtained data were used for evaluation of phenolic compound effect on MEs characteristics.

Thermodynamic stability studies demonstrated that o/w MEs were stable in centrifugation and heating-cooling tests although minor instability was determined in freeze-thaw testing.

The measurements of pH, conductivity, and viscosity were performed 24 hours after formulating o/w MEs [18] to detect possible effects of ethanolic propolis extract (Table 3). The results confirmed absence of critical effects of propolis phenolic compounds on the physical characteristics of MEs.

The pH and viscosity of the MEs were not significantly (*P* > 0.05) affected by changing ethanol to ethanolic propolis extract. The conductivity of the MEs containing ethanolic propolis extract was similar to the MEs containing ethanol thus confirming the same type of o/w dispersion.

All o/w microemulsions were characterized measuring their droplets size and polydispersity index (PDI) (Table 4) and determining number of particle fractions in MEs. The measurements of droplet size in MEs after 24 hours and 1 week were performed to evaluate possible changes of nanostructured systems.

Mean droplet size of MEs containing ethanol or ethanolic propolis extract after 24 hours was similar to the values determined after 1 week. The increase of the surfactant-cosurfactant ratio in MEs resulted in increased mean droplet size but had no effect on the PDI of the MEs. Two-peak particle distribution pattern was determined for MEs formulated with S-CoS ratio 2 : 1; thus it was not considered for further development of ME formulation. Propolis microemulsion with S-CoS weight ratio 3 : 1 and containing 5% isopropyl myristate as oily phase was selected as offered maximum quantity of ethanolic propolis extract to be incorporated. The evaluation of oily phase amount effect on the physical parameters and stability of MEs was performed variating isopropyl myristate content from 3 to 7% (Table 5).

The values of mean droplet size of o/w propolis MEs containing 3%, 5%, or 7% of isopropyl myristate 1 week after formulation did not differ from values determined 24 hours after formulation. Single peak droplet distribution was identified for the MEs containing 7% of isopropyl myristate while MEs with 3% and 5% concentration of isopropyl myristate contained droplets of two size fractions.

Biopharmaceutical characterization of MEs containing 7% of isopropyl myristate was performed evaluating penetration of *p*-coumaric, ferulic, caffeic, vanillic acids, and vanillin into skin layers* ex vivo*. No propolis polyphenolic compounds were determined in epidermis after 24 hours of application of ME, and only vanillin and *p*-coumaric acid were determined in dermis. Essential oils of pine needle and spruce needle were added to o/w propolis microemulsions to improve the penetration of phenolic compounds into the skin [19]. The MEs contained 1% of the essential oil decreasing the concentration of isopropyl myristate, respectively. The propolis MEs were characterized measuring their droplets size and PDI and number of peaks after 24 hours and 1 week (Table 6). The mean droplet size and PDI were not affected by the added essential oil and did not change after 1 week.

The determined fluxes of phenolic compounds into human epidermis and dermis from propolis MEs containing 7% of isopropyl myristate and chemical penetration enhancers are presented in Table 7. Inclusion of pine needle essential oil or spruce needle essential oil resulted in no significant increase of vanillic, caffeic,* p*-coumaric, ferulic acids, and vanillin in epidermis. Only* p*-coumaric acid was identified but the quantity was below limit of quantification. The increased amounts of* p*-coumaric, ferulic acids, and vanillin were determined in dermis with the addition of essential oils, and their increase was higher when ME contained 1% spruce needle essential oil. The presence of vanillic and caffeic acids was determined in dermis but amounts their quantities were below limit of quantification.

Low penetration of propolis phenolic compounds from MEs could be explained by the possible distribution pattern of vanillic, caffeic,* p*-coumaric, ferulic acids, and vanillin in the MEs as due to their solubility they could be concentrated in the interfacial film, formed by surfactant and cosurfactant as well as in the oily phase of MEs [9, 13]. Thus the availability of phenolic compounds in the external phase of the MEs for absorption into the skin can be a limiting factor for their penetration into skin. The addition of essential oils to the MEs increased the amounts of phenolic compounds penetrating into skin, but the increase was not statistically significant. Only in case of ferulic acid did the quantified amount in dermis after application of ME with 1% spruce needle essential oil increase significantly.

## 4. Conclusion

Propolis phenolic compounds are considered as potent antioxidants that could be applied for minimization of deleterious effects of oxidative stress on biological systems of the living organisms. The important prerequisite for achieving desirable biological effect is availability of propolis phenolic compounds at the site of possible oxidative damage. Therefore the techniques to increase intradermal penetration of phenolic compounds are attracting much interest. Application of MEs could result in increased quantities of phenolic compounds in skin layers due to presence of relatively high amounts of surface active agents and their ability to disturb the lipid matrix structure and resultant increased permeability of the skin. The results of demonstrated limited ability of MEs formulated using PEG-8 caprylic/capric glycerides and ethanolic propolis extracts to improve delivery phenolic compounds into skin* ex vivo*. The inclusion of 1% of pine needle or spruce needle essential oils into MEs produced no effect on the droplet size and polydispersity index, and this may indicate that the components of essential oils were concentrated in oily phase; hence their potential effect on the skin barrier was limited. Considering limited penetration of propolis phenolic compounds into the skin in the* ex vivo* testing, the efforts should be made to formulate MEs able to incorporate higher quantities of propolis extract. The compositions of MEs had to be optimized to achieve higher concentrations of propolis phenolic compounds in the external phase thus ensuring the availability of biologically active compounds for penetration into biological membranes.

## Figures and Tables

**Table 1:** Compositions of oil-in-water propolis microemulsions.

Ratio of S : CoS	2 : 1	3 : 1	4 : 1	5 : 1	6 : 1	7 : 1	8 : 1	9 : 1
Isopropyl myristate (%)	5.0	5.0	5.0	5.0	5.0	5.0	5.0	5.0
Purified water (%)	25.0	25.0	25.0	25.0	25.0	25.0	25.0	25.0
Labrasol (%)	46.7	52.5	56.0	58.3	60.0	61.3	62.2	63.0
Ethanolic propolis extract (%)	23.3	17.5	14.0	11.7	10.0	8.8	7.8	7.0

**Table 2:** Quantitative profile of phenolic compounds in Lithuanian propolis extracts.

Propolis extract type	Phenolic compound ± SD (*µ*g/mL)				
	Vanillic acid	Caffeic acid	Vanillin	*p*-Coumaric acid	Ferulic acid
Ethanolic	267.7 ± 23.5	59.0 ± 4.4	507.3 ± 11.2	983.7 ± 8.0	654.9 ± 4.1
Aqueous	56.1 ± 3.8	10.2 ± 2.0	80.6 ± 3.0	119.6 ± 2.8	64.1 ± 2.0

**Table 3:** Characteristics of o/w microemulsions containing ethanolic propolis extract and controls.

Ratio of S : CoS	pH		Conductivity (*µ*S/cm)		Viscosity (mPa·s)	
	ME with EtOH	ME with propolis extract	ME with EtOH	ME with propolis extract	ME with EtOH	ME with propolis extract
2 : 1	4.79	4.60	10.4	10.8	11.2	13.2
3 : 1	4.72	4.57	9.7	9.9	14.7	17.8
4 : 1	4.68	4.56	9.3	9.4	18.5	22.1
5 : 1	4.64	4.56	9.1	9.0	22.1	24.4
6 : 1	4.59	4.55	8.8	8.8	26.9	27.7
7 : 1	4.58	4.54	8.6	8.6	29.1	29.6
8 : 1	4.57	4.52	8.5	8.5	31.0	32.3
9 : 1	4.56	4.52	8.4	8.3	31.2	36.4

**Table 4:** Mean droplet size and PDI of o/w microemulsions.

Ratio of S : CoS	Mean droplet size ± SD (nm)/PDI			
	24 hours		1 week	
	ME with EtOH	ME with ethanolic propolis extract	ME with EtOH	ME with ethanolic propolis extract
2 : 1	34.95 ± 0.19	43.69 ± 0.41	34.97 ± 0.17	43.49 ± 0.46
	0.299 ± 0.003	0.274 ± 0.020	0.300 ± 0.005	0.268 ± 0.017
3 : 1	40.67 ± 0.32	48.83 ± 0.31	40.45 ± 0.35	48.62 ± 0.42
	0.310 ± 0.004	0.274 ± 0.006	0.304 ± 0.003	0.285 ± 0.020
4 : 1	46.80 ± 0.86	55.30 ± 0.45	46.23 ± 0.29	55.42 ± 0.36
	0.312 ± 0.005	0.285 ± 0.022	0.312 ± 0.005	0.275 ± 0.008
5 : 1	51.53 ± 0.56	62.10 ± 0.39	51.34 ± 0.35	62.17 ± 0.54
	0.312 ± 0.002	0.271 ± 0.009	0.305 ± 0.008	0.272 ± 0.007
6 : 1	57.10 ± 0.43	66.24 ± 0.41	57.32 ± 0.17	66.45 ± 0.50
	0.309 ± 0.004	0.271 ± 0.007	0.307 ± 0.009	0.271 ± 0.005
7 : 1	61.85 ± 0.41	72.02 ± 0.62	62.07 ± 0.10	72.15 ± 0.53
	0.299 ± 0.004	0.266 ± 0.006	0.299 ± 0.009	0.269 ± 0.009
8 : 1	65.88 ± 0.63	79.58 ± 1.07	66.78 ± 0.62	77.50 ± 0.15
	0.300 ± 0.003	0.288 ± 0.021	0.295 ± 0.002	0.268 ± 0.003
9 : 1	69.56 ± 0.68	80.24 ± 1.12	70.26 ± 0.85	80.68 ± 0.13
	0.292 ± 0.003	0.274 ± 0.011	0.289 ± 0.005	0.267 ± 0.002

**Table 5:** Characteristics of propolis microemulsions containing different concentrations of oily phase.

Isopropyl myristate (%)	Mean droplet size (nm)		PDI	
	24 hours	1 week	24 hours	1 week
3	41.69 ± 0.26	41.29 ± 0.30	0.291 ± 0.023	0.299 ± 0.017
5	48.83 ± 0.31	48.62 ± 0.42	0.274 ± 0.006	0.285 ± 0.020
7	60.53 ± 0.32	60.84 ± 0.58	0.248 ± 0.007	0.254 ± 0.008

**Table 6:** Characteristics of propolis microemulsions containing 1% of essential oil.

Essential oil	Mean droplet size (nm)		PDI	
	24 hours	1 week	24 hours	1 week
Pine needle	57.08 ± 0.43	57.28 ± 0.44	0.256 ± 0.009	0.253 ± 0.006
Spruce needle	57.19 ± 0.36	57.11 ± 0.28	0.246 ± 0.008	0.251 ± 0.005

**Table 7:** The fluxes of phenolic compounds from propolis microemulsions into skin layers after 24 hours.

Phenolic compounds	Flux (*µ*g/cm^2^)		
	PME (3 : 1) containing 7% IMP	PME (3 : 1) containing 6% IMP and 1% pine needle essential oil	PME (3 : 1) containing 6% IMP and 1% spruce needle essential oil
Epidermis			
Vanillic acid	BLD	BLD	BLD
Caffeic acid	BLD	BLD	BLD
Vanillin	BLD	BLD	BLD
*p*-Coumaric acid	BLD	ALD	ALD
Ferulic acid	BLD	BLD	BLD
Dermis			
Vanillic acid	ALD	BLD	ALD
Caffeic acid	BLD	BLD	ALD
Vanillin	0.43 ± 0.10	0.49 ± 0.13	0.62 ± 0.14
*p*-Coumaric acid	0.28 ± 0.10	0.34 ± 0.11	0.36 ± 0.10
Ferulic acid	ALD	ALD	0.35 ± 0.12

ALD: above limit of detection.

BLD: below limit of detection.
